# Sulfide-Based All-Solid-State Lithium–Sulfur Batteries: Challenges and Perspectives

**DOI:** 10.1007/s40820-023-01053-1

**Published:** 2023-03-28

**Authors:** Xinxin Zhu, Liguang Wang, Zhengyu Bai, Jun Lu, Tianpin Wu

**Affiliations:** 1 College of Chemical and Biological Engineering, Zhejiang University, Hangzhou, 310058 People’s Republic of China; 2 Collaborative Innovation Center of Henan Province for Green Manufacturing of Fine Chemicals Key Laboratory of Green Chemical Media and Reactions Ministry of Education, School of Chemistry and Chemical Engineering, Henan Normal University, Xinxiang, Henan 453007 People’s Republic of China

**Keywords:** All-solid-state lithium–sulfur battery, Sulfur cathode, Triple-phase interfaces, Electrolyte decomposition, Volume change

## Abstract

Lithium–sulfur batteries with liquid electrolytes have been obstructed by severe shuttle effects and intrinsic safety concerns. Introducing inorganic solid-state electrolytes into lithium–sulfur systems is believed as an effective approach to eliminate these issues without sacrificing the high-energy density, which determines sulfide-based all-solid-state lithium–sulfur batteries. However, the lack of design principles for high-performance composite sulfur cathodes limits their further application. The sulfur cathode regulation should take several factors including the intrinsic insulation of sulfur, well-designed conductive networks, integrated sulfur-electrolyte interfaces, and porous structure for volume expansion, and the correlation between these factors into account. Here, we summarize the challenges of regulating composite sulfur cathodes with respect to ionic/electronic diffusions and put forward the corresponding solutions for obtaining stable positive electrodes. In the last section, we also outlook the future research pathways of architecture sulfur cathode to guide the develop high-performance all-solid-state lithium–sulfur batteries.

## Highlights

- The composite cathode composition, preparation method, and chemical compatibility play critical roles in constructing triple-phase interfaces.
- Understanding the electrolyte degradation is critical for boosting the high-performance composite sulfur cathode.
- The volume change of sulfur challenges the mechanical stability of composite sulfur cathode.

## Graphical abstract

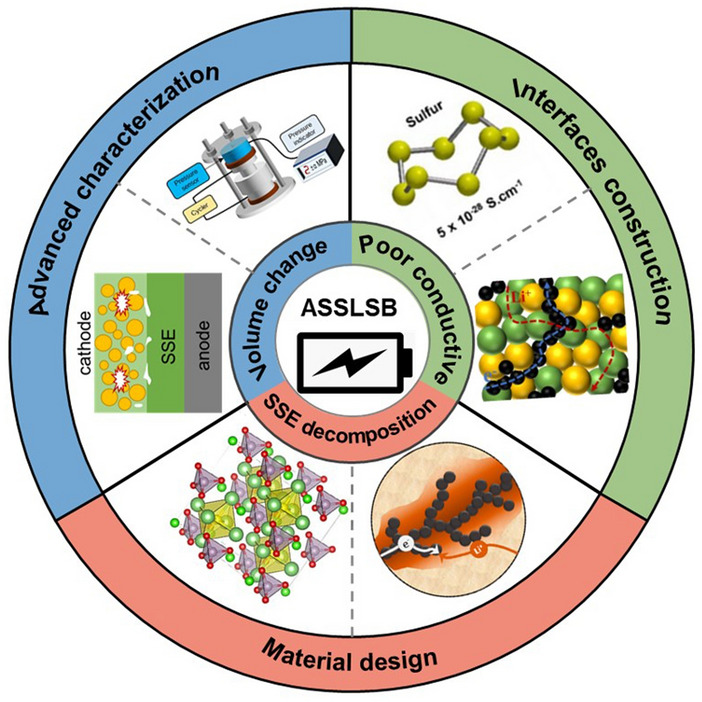

## Introduction

Lithium–sulfur (Li–S) batteries have drawn significant interest owing to the high theoretical capacity of both-side electrodes (Li: 3,860 mAh g^−1^; S: 1,675 mAh g^−1^) [1–3]. Unfortunately, the shuttle effect of the intermediate polysulfides has hampered the development of liquid Li–S batteries [4, 5]. These polysulfides formed during the sulfur reaction are highly soluble in liquid electrolytes and can transfer to the anode side through the electrolytes, resulting in the loss of active materials and low coulombic efficiencies [6, 7]. Although this issue can be suppressed by trapping sulfur through designing carbon scaffolds, replacing liquid electrolytes with solid-state electrolytes is the most promising alternative strategy to eliminate it [8–10]. Recently, various solid-state electrolytes have been developed including polymer electrolytes, oxide electrolytes, halide electrolytes, and sulfide electrolytes. The polymer electrolytes generally exhibit poor ionic conductivity at room temperature and limited effects on suppressing the intrinsic issue of “shuttle effect” in lithium–sulfur batteries. For oxide-based solid-state electrolytes, the mechanical stiffness is unfavorable for composite sulfur cathode manufacturing. For halide electrolytes, although these materials possess the advantages of high ionic conductivity and high-voltage stability, the (electro–) chemical reactions between halide electrolytes and lithium metal anodes are discerned as the foremost drawback [12]. Sulfide solid-state electrolytes reserve the highest ionic conductivity among these solid electrolytes and are analogs to sulfur cathode determining good chemical compatibility. Additionally, SSEs generally demonstrate the mechanically soft property which allows for cold-pressing to secure dense and intimate physical contacts between composite cathode components, which benefits practical electrode regulation [13–15]. These features adjudicate SSEs as the most promising solid-state electrolyte for all-solid-state lithium–sulfur batteries (ASSLSBs) [16, 17].

Despite the promising benefits, there are still several remaining challenges toward practical ASSLSBs, especially on cathode electrodes which are highly correlated to the electrochemical properties [18, 19]. One primary issue is that the insulation of active materials sulfur requires composite cathode formation with solid-state electrolytes and conductive agents to gain sufficient ions and electrons at trip-phase interfaces [20, 21]. To obtain composite cathodes with good physicochemical properties, these multiple components need to be chemically compatible and to be well-regulated for building uniform conductive pathways [22]. Apart from exploring the preparation method and the compatibility among these components, the compositing process itself makes the carrier transport pathways extremely tortuous [23]. While increasing active material loading can boost the energy density, long diffusion pathways accompanied by sluggish ion transport result in large electrochemical polarization and low specific capacity. Therefore, it is critical to carefully balance carrier transport and energy density by regulating the composition of the composite sulfur cathode. Another issue is that severe degradation of the SSEs usually occurs and forms inactive productions because of poor electrochemical stability and substantial contact with conductive agents, hindering ionic/electronic transport, and thus degrading the electrochemical properties [24, 25]. In addition to the specific phenomena in solid-state battery systems, the intrinsic large volume change of sulfur originating from the conversion reaction usually can break the physical contact, dramatically reducing the conductive pathways [26]. Furthermore, these challenges are always symbiotic and interconnected with each other, severely limiting the development of practical ASSLSBs. The sluggish reaction kinetics can be further worsened by electrolyte degradation and the formation of cracks. Therefore, it is essential to summarize the abovementioned challenges to provide a fundamental understanding of composite sulfur cathode and guide the future design of sulfide-based ASSLSBs.

Herein, in this perspective, we overview the challenges for developing high-performance composite sulfur cathodes. The correlated fundamental understanding and possible strategies to solve the corresponding challenge are also highlighted. In the last section, we also give our outlook on the design of composite sulfur cathodes for the further development of ASSLSBs. This perspective provides a fundamental understanding of next-generation high-energy and safe sulfide-based ASSLSBs.

## Challenges and Solutions for the Composite Sulfur Cathode

Despite sulfide-based ASSLSBs demonstrating great potential for high energy and safety, the chemical properties of SSEs such as air stability brings many challenges to the practical application of batteries. The SSEs are generally highly sensitive to moisture in the air, leading to the production of toxic H_2_S gas and structural degradation of electrolytes. This is attributed to the weak P–S bond energy in SSEs, making phosphorus tend to bond with oxygen in the moisture environment, which can be well explained by the hard and soft acid–base theory. Several effective strategies have been proposed in the design of air-stable SSEs including the use of H_2_S absorbents, elemental substitution, surface engineering, and the development of new materials [27]. In addition to the chemical stability of the SSEs, the introduction of SSEs into sulfur cathodes challenges the ionic/electronic transport design in positive electrodes. The composite sulfur cathodes in the sulfide-based ASSLSBs generally consist of elemental sulfur, sulfide solid-state electrolytes, and conductive materials (e.g., carbon black). Both ionic and electronic diffusion behavior depend on solid–solid interfaces among these components, which determines the electrochemical properties of the whole battery. To realize high-performance composite sulfur cathodes, constructing stable tripe-phase interfaces with good electrochemical kinetic is essential, which requires increasing the conductivity of active materials, suppressing electrolyte decomposition, and inhibiting the volume changes of sulfur during cycling.

### Sluggish Reaction Kinetics of Active Materials

**Fig. 1:**
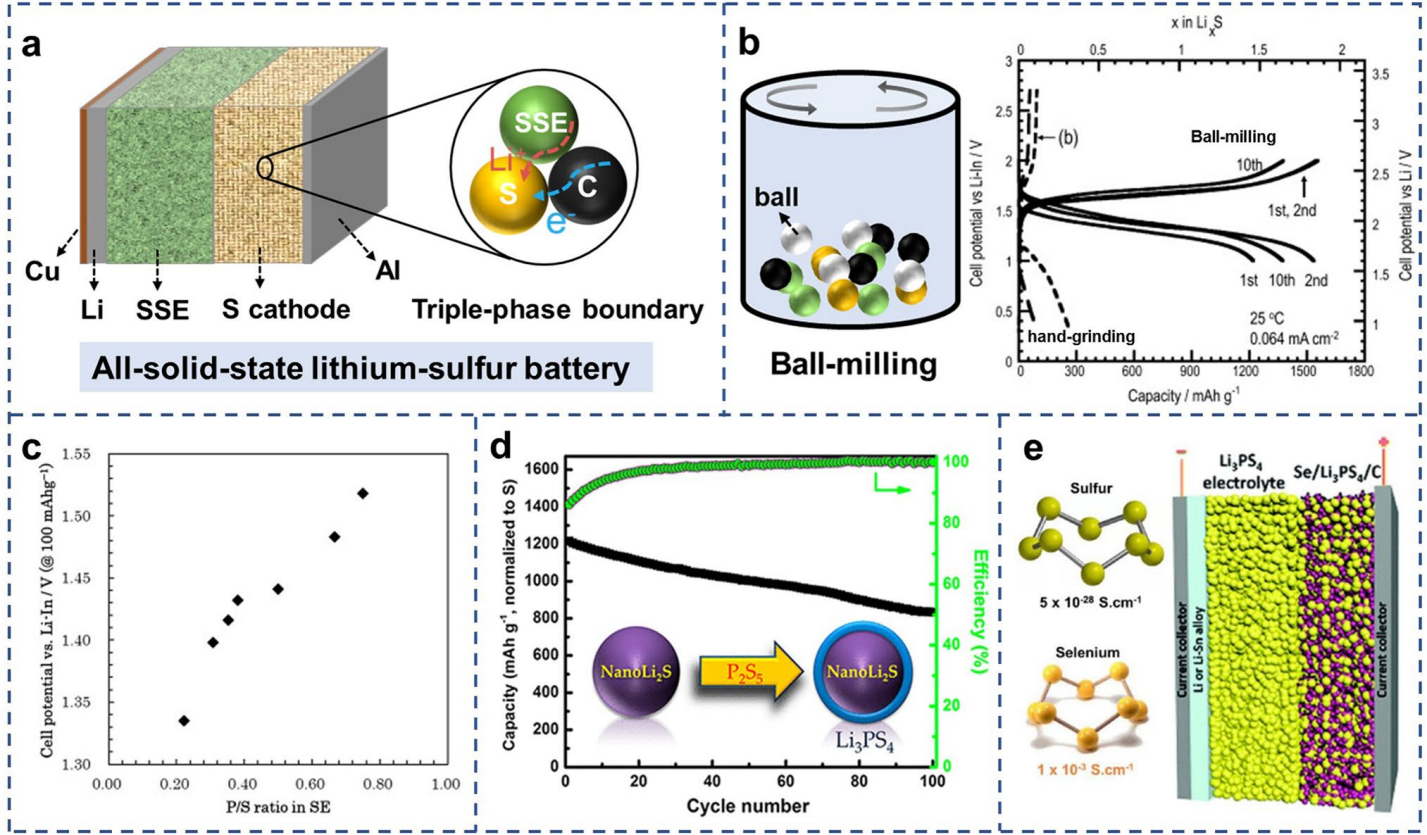
**a** Schematic illustration of all-solid-state lithium–sulfur battery. **b** Charge–discharge curves of all-solid-state cells of Li–In/0.8Li_2_S·0.2P_2_S_5_/S using hand-grinding and ball-milling. Copyright from Ref. [28]. **c** Plots of the discharge potentials for various P/S ratios in SSEs. Copyright from Ref. [32]. **d** Electrochemical evaluation of NanoLi_2_S as the cathode material for ASSLSBs at 60 ℃. Copyright from Ref. [35]. **e** Illustration of all-solid-state Li-Se batteries highlighting the significantly higher electronic conductivity of selenium compared to that of elemental sulfur. Copyright from Ref. [37]

The ionic and electronic insulation of active material sulfur brings great complexity to designing high-performance composite sulfur cathodes. This inherent drawback requires preparing sulfur cathodes by combining sulfur with solid-state electrolytes and electronic conductive additives to build the connection networks and triple-phase boundaries (Fig. 1a), resulting in tortuous and long ionic/electronic transport pathways [11]. To achieve superior reaction kinetic in the composite sulfur cathode, a sufficient supply of ions and electrons at the triple-phase interfaces is required. However, since increasing electrolytes and conductive carbon facilitates the ion and electron diffusion kinetics, it sacrifices attainable energy density due to these introduced inactive masses. Therefore, an optimization of the mixing ratio among components that enables the highest possible active material loading is needed. Moreover, the ionic diffusion kinetic in the composite sulfur cathode is closely associated with the operating temperature and evolves worse at low temperature. To enhance ionic diffusion in the all-climate operating environment, increasing the ionic conductivity of electrolytes is believed as an effective strategy. Kanno’s group developed a series of SSEs that demonstrate the higher ionic conductivity of 2.5 × 10^−2^ S cm^−1^ at room temperature than that of commercial liquid electrolytes (1.0 × 10^−2^ S cm^−1^) [15]. Even at a temperature of 0 °C, the ionic conductivities of these SSEs still remain 1–5 mS cm^−1^, eliminating the concerns caused by the low ionic diffusion kinetics in common solid-state electrolytes. However, to achieve superior ionic kinetic in an all-climate operating environment, stable and dense solid–solid interfaces that provide generous diffusion pathways are required.

The multiple components of the composite cathode challenge the preparation of a cathode with uniform physicochemical properties. To this end, the fabrication process plays a critical role in constructing triple-phase interfaces based on the uniform distribution of each component. Compared to the typical mixing method by hand-grinding, mechanical ball-milling can decrease the particle size of all components to uniform mixing, which increases the contact area of tripe-phase interfaces within composite sulfur cathodes. The S-SSEs-C composites prepared by ball-milling methods can deliver high specific capacities at the first cycle (Fig. 1b) and exhibit a long cycle life [28]. On this basis, high-temperature ball-milling technology was developed to fabricate cathode materials [29]. At the high temperature of 155 °C, the sulfur displays low viscosity, which facilitates its sublimating into the void of conductive mediums. Further coupling with the ball-milling treatment, all components can be uniformly distributed, achieving high-performance homogenous composite cathodes. Other various precursors mixing methods such as liquid-phase and gas-phase mixing are also developed to build stable triple-phase interfaces, while these methods usually require complex processing leading to high-energy consumption or high cost [30, 31].

Apart from the fabricating technology, the selection of electron/ion additives, especially when considering electrochemical compatibility, is essential for acquiring stable interfaces and good properties [11, 32]. For instance, a positive correlation between the P/S ratio in SSE and the reactivity of sulfur was revealed in the literature [32]. Compared to other SSEs, despite the lower ionic conductivity, sulfide electrolytes Li_1.5_PS_3.3_ with a higher P/S ratio exhibit suppressed voltage polarization and high initial specific capacity (Fig. 1c) [32]. This is because the sulfur element reacts with phosphorus in Li_1.5_PS_3.3_ forming phosphorus sulfides besides transforming to Li_2_S, which indicates the high chemical reactivity of sulfur. The relationship between different components is also revealed to determine the overall physical and electrochemical properties of the composite sulfur cathode [33]. The surface area of conductive material is found to have a greater influence on battery performance than electronic conductivities. Among carbon-dotted materials, composite sulfur cathode containing activated carbon with a high surface area can deliver extremely larger reversible capacities than other carbon materials including acetylene black and Ketjenblack.

There are additional approaches to reduce the detrimental effects raising from the intrinsic insulation of sulfur and improve ion or electron conduction. Replacing the elemental sulfur with lithium sulfide material as active material benefits the reaction kinetics of the composite cathode due to the higher ionic conductivity (10^–13^ S cm^−1^) than that of sulfur (10^–30^ S cm^−1^) [34]. The electrochemical reaction of S and Li_2_S cathodes are reversible and both are based on the conversion reactions of Li + S = Li_2_S. Especially, Li_2_S can be employed as both active material and the basic component of SSEs. Together with other components such as P_2_S_5_, it can in situ generate SSEs on the surface of Li_2_S particles [35, 36]. As shown in Fig. 1d, the Li_2_S@SSE core–shell structure simplifies the triple-phase boundaries, improving the ionic transport coefficient inside the composite cathode. In addition to employing Li_2_S, introducing metal elements into sulfur cathode to form MS_x_ (M = Se, Fe, Mo, etc.) solid solutions is another strategy to significantly improve the electrochemical reaction kinetics and active material utilization (Fig. 1e) [37, 38]. Although part of energy density is sacrificed through this strategy, fast carrier transport and good rate capability are obtained. Based on the abovementioned knowledge, fundamental insights into interface architecture are critical for boosting the electrochemical properties of ASSLSBs.

### Electrolyte Decomposition

**Fig. 2:**
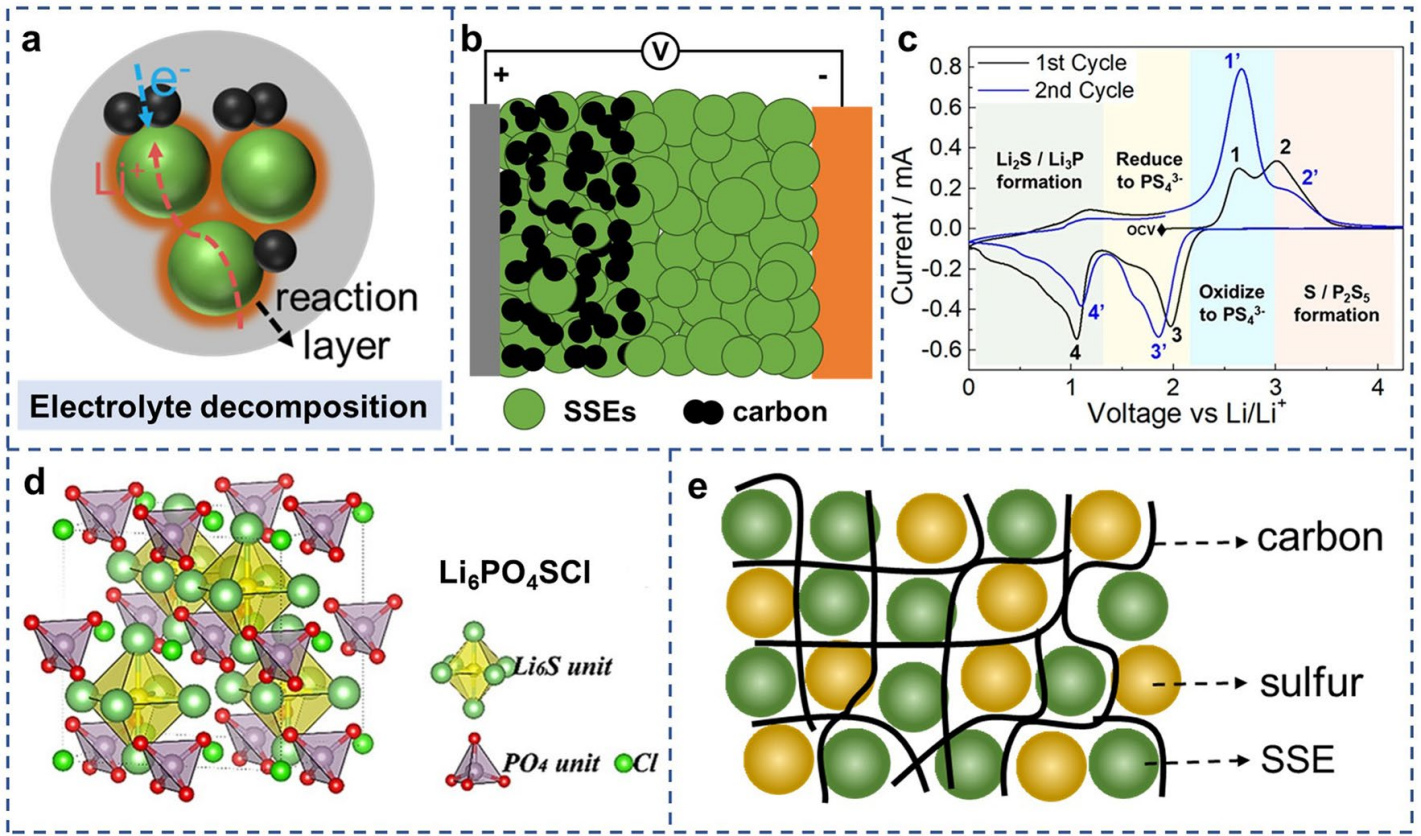
Schematic diagram of **a** SSEs decomposition in ASSLSBs and **b** all-solid-state battery using SSEs-C electrodes. **c** Cyclic voltammograms of Li − In/Li_6_PS_5_Cl /Li_6_PS_5_Cl-C half-cell. Copyright from Ref. [25]. **d** The structural model of Li_6_PO_4_SCl. Copyright from Ref. [48]. **e** Composite sulfur cathodes consist of sulfur, SSEs, and multi-dimensional carbon

Electrolyte decomposition has a significant effect on the interfacial physicochemical properties at the triple-phase interfaces. Although the cut-off voltage of 2.8 V for the composite sulfur cathode is lower than most high-voltage positive electrodes such as LiCoO_2_, the application of a large number of conductive carbons in the composite cathode accelerates the decomposition of SSEs [39]. The SSEs undergo a redox reaction at the SSEs-C interfaces during cycling, resulting in electrochemically inert products (Fig. 2a). These degradation products are always sticking to the vicinity of triple-phase boundaries, which damages ionic/electronic diffusion networks in the composite cathode, and thereby, leading to severe voltage polarization and fast capacity decay [23].

Understanding the electrochemical stability of electrolytes is essential for designing stable composite sulfur cathodes. The thermodynamic stable electrochemical window of solid-state electrolytes can be studied by first-principles calculations. Professor Mo has calculated the voltage window for most SSEs, such as 1.71–2.14 V and 1.71–2.01 V vs Li/Li^+^ for Li_10_GeP_2_S_12_ and Li_6_PS_5_Cl, respectively, which provides guidance for the design of electrochemically stable electrolytes [40]. Recent studies reported the electrochemical stability of electrolytes can be evaluated by using Li/SSEs/SSEs-C cell configuration (Fig. 2b) [25, 41]. Combining electrolytes with conductive materials could increase the overall electronic conductivity of electrodes, which was beneficial for amplifying the decomposition current signal and reflecting the intrinsic electrochemical windows of sulfide electrolytes. By galvanostatic tests, the large capacity of SSEs-C electrodes at the first cycle demonstrates that SSEs suffer decomposition reactions. This is the reason that the coulombic efficiency of several ASSLSBs exceeds 100%. The specific capacity value is highly related to the physical and chemical properties of materials, the mass fraction of SSEs, and the operating temperature. On extended cycling, the capacity remains relatively constant, which could indicate that the decomposition products are able to deliver reversible electrochemical activity [25]. However, the degradation also influences the overall transport within the composite cathode and the cell cyclability. With the cycling voltammetry characterization of the Li/SSEs/SSEs-C cell, the detailed decomposition process and resulting products can be revealed (Fig. 2c) [41]. It is of great significance to simulate and characterize SSEs-C electrodes in the operating environment of the composite sulfur cathode, linking ion transport in a composite to the electrolyte degradation and ASSLSBs cycling performance, which is beneficial to understand and weaken the influence of electrolyte decomposition on the electrochemical performances of cells.

There are two main strategies to reduce the (electro–) chemical decomposition of SSEs in the composite sulfur cathode. One is to expand the voltage window of the SSEs [42, 43], and the other is employing specific conductive agents to reduce the contact areas with the electrolytes [44]. Element doping such as Cl, Nb, and O is an effective way to improve the electrochemically stabilities of SSEs (Fig. 2d) [45–48]. The composite sulfur cathode with elemental doping can suppress interfacial side reactions, which improves the overall electrochemical performance of ASSLSBs. In addition, multi-dimensional carbon materials with a high aspect ratio as electronic conductive additives are proven to reduce the decomposition of electrolytes, achieving the stable long-cycling of ASSLSBs (Fig. 2e) [9]. This is because these carbon materials demonstrate a low surface area compare to conventional carbon-dotted particles, which reduces the possibility of physical contact with electrolytes, thereby weakening the redox reactivity of electrolytes. Besides, this multi-dimensional material can also provide a fast-conductive network that is beneficial for the electrochemical reaction kinetics.

### Volume Change

**Fig. 3:**
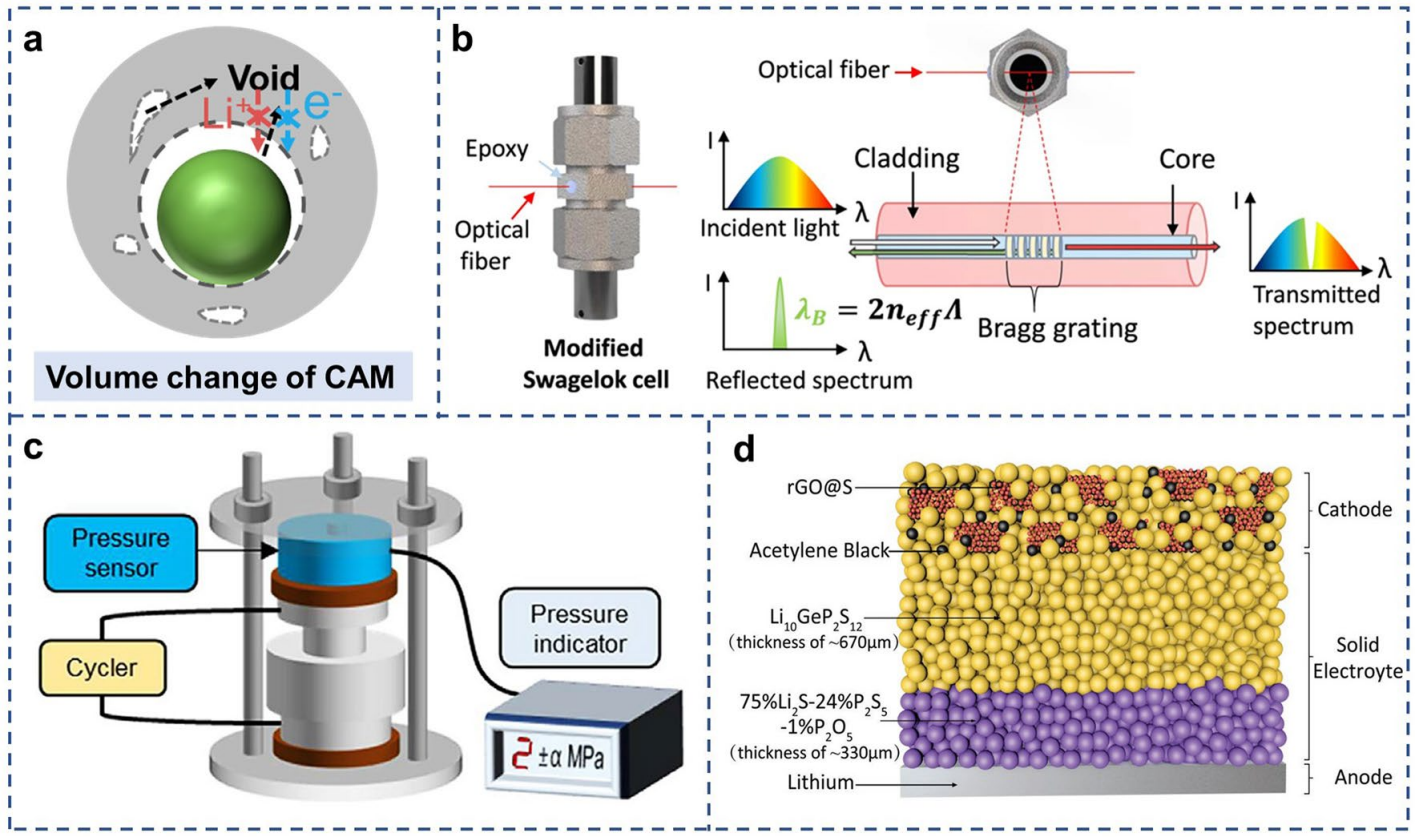
**a** Schematic diagram of volume change in the composite sulfur cathode. **b** Scheme of the integration of an FBG into an in-house modified Swagelok cell together with the working principle of an FBG optical sensor. Copyright from Ref. [52].**c** Schematic diagram of pressure monitoring all-solid-state cells. Copyright from Ref. [53]. **d** Schematic diagram of all-solid-state lithium–sulfur battery using rGo@S composite cathode

Volume changes associated with (de)lithiation of active materials can cause mechanical fracture such as the formation of cracks, decreasing the battery cycling stability [49]. The electrode materials experiencing conversion chemistry such as sulfur usually produce large volume expansion. The sulfur cathodes with the chemical reaction of 16Li + S_8_ ↔ 8Li_2_S exhibit about 80% volume changes compared to the pristine sulfur when lithiated to Li_2_S [50]. However, the rigid solid-state electrolytes are unable to accommodate the volume change of the element sulfur, thus resulting in the build-up of stress inside the composite cathode. The mechanical fracture will occur including the formation of cracks upon long-term cycling (Fig. 3a). More seriously, the volume change in the positive electrodes can pass on to other battery components including electrolyte layers, which could induce severe mechanical failure at electrolyte/electrode interfaces, directly degrading the life span. For eliminating this issue, researchers usually utilize high external press to enhance the physical interfacial contact between different components. Unfortunately, mechanical failures caused by severe volume changes still exist, limiting large-scale practical applications at low external pressure.

To better understand the mechanical evolutions originating from large volume changes of the sulfur cathode, it is essential to develop one technique to measure the corresponding internal stress evolution during cycling. Generally, there is a relationship between the volume changes of the active materials and the pressure change of the bulk battery [51]. The actual internal pressure change of the cell can be investigated by pressure sensors as reported in the literature. At present, the two primary types of stress measurement devices employed in sulfide-based all-solid-state lithium metal batteries are the built-in optical Fiber Bragg Grating (FBG) sensor and the external pressure sensor. The former introduces an integrated optical FBG sensor into the battery system to measure the pressure by converting the Bragg wavelength and the corresponding physical parameters of the FBG sensor (Fig. 3b) [52]. The latter applies an external pressure sensor outside the battery system to detect the overall stress change of the whole cell during cycling (Fig. 3c) [49, 53]. The two methods differ in many aspects in terms of the operational complexity, the degree of damage to the material, and the direction of the obtained pressure [54]. To accurately evaluate the volume change in the composite sulfur cathode, employing zero-strain anodes like L_4_T_5_O_12_ (LTO) as the counter electrode seems to be an effective way to eliminate the effect of large volume changes of lithium anodes [53]. This LTO-S equipment combined with pressure sensors outside the cell can continuously obtain evolution regularity of the volume inside the positive electrode during the cycle, which provides a powerful tool for investigating the relationship between mechanical effects and battery electrochemical performance.

Based on the abovementioned knowledge, several strategies are proposed in the literature to deal with the mechanical failures in the composite sulfur cathode. Typically, various sulfur/carbon structures are fabricated to withstand the volume change during the discharge and charge processes [55–57]. The introduction of carbon holes or tubes can provide large spaces to accommodate the expansion and contraction of sulfur. For example, the combination of S and rGo with ultrahigh electronic conductivity and large surface area exhibits excellent mechanical properties, which can alleviate the negative impacts of volume change of sulfur cathode (Fig. 3d) [55]. Notably, a series of sulfur/carton structures have been developed for liquid Li–S batteries, but few of them work well for the corresponding solid-state systems in ASSLSBs. This is because the complex sulfur/carbon structures such as “tube in tube” and “nest” in the liquid are mainly designed to rivet sulfur and reduce the dissolution of the polysulfides, which may block ionic transport in ASSLSBs due to the difference in fluidity between solid electrolytes and liquid electrolytes [58, 59]. Moreover, the introduction of polymers in electrodes to bind the components together is an effective strategy for improving mechanical stability. The binder owning large elasticity can be mobile as the volume change of the active materials, avoiding the contact failure between electrode components [60]. However, the additional application of binders in the composite cathode preparation can also increase the complexity of the electrode manufacturing process. Considering the dispersion of binders, wet-slurry-based manufacturing methods using the polar solvent can completely dissolve polymeric binders to achieve uniform distribution of the components [61]. Nevertheless, many SSEs are sensitive to polar organic solvents for slurry preparation, exhibiting a series of negative effects such as dissolution, complexation, and degradation, which could reduce the ionic conductivity of electrolytes and increase cell impedance. Therefore, adjusting the compatibility among SSEs, binder, and solvent is the key to applying the wet-slurry process. Dry-processed electrode technique employing fibrous binders renders the electrodes form films under the action of shear force, which exhibits unique advantages compared to the conventional wet-slurry methods due to the non-solvent process that includes enhanced compatibility, environmental friendliness, and improved electrode performances. However, a limited number of polymer types such as PTFE binder are suitable for this method [62, 63]. Additionally, developing mechanically flexible electrolytes that can accommodate volume changes of sulfur could be another effective strategy to gain highly mechanically stable ASSLSBs [64, 65].

## Perspectives

In this perspective, we summarize the main challenges focusing on the design principles of sulfur cathode electrodes for ASSLSBs. A comprehensive understanding of currently facing issues with the composite sulfur cathode and the corresponding strategies to achieve advanced high-performance cathodes are presented. Following these researches, to realize durable ASSLSBs, more efforts are still required with respect to several aspects [66, 67, 68]. Firstly, tremendous efforts in building fast ionic/electronic transport pathways are of great importance for boosting electrochemical performance, especially for the high-loading cathodes in practical applications. Secondly, developing novel solid-state electrolytes with high ionic conductivities and good electrochemical stability is helpful for enhancing ionic diffusion and reducing side reactions with other components in the composite cathode. Thirdly, exploring ASSLSBs operating at low external pressure can put a great step forward to practical applications. Last but not the least, leveraging advanced in situ/in operando characterization techniques to reveal fundamental electrochemical reaction and degradation mechanisms of composite sulfur cathodes is another strategy to guide further advanced cathode design for high-energy and safe ASSLSBs.
